# A data plane security model of segmented routing based on SDP trust enhancement architecture

**DOI:** 10.1038/s41598-022-12858-2

**Published:** 2022-05-24

**Authors:** Liang Wang, Hailong Ma, Yiming Jiang, Yin Tang, Shuodi Zu, Tao Hu

**Affiliations:** 1 Institute of Information Technology, PLA Strategic Support Force Information Engineering University, Zhengzhou, 450003 China; 2 Cyberspace Security College, PLA Strategic Support Force Information Engineering University, Zhengzhou, 450003 China; 3 National Digital Switching System Engineering Technology Research Center, Zhengzhou, 450003 China; 4 Network Communication and Security Purple Mountain Laboratory, Nanjing, 211111 China

**Keywords:** Electrical and electronic engineering, Computer science, Information technology

## Abstract

Segment routing (SR) technology is a new network functional technology derived from MPLS technology and based on SDN. Combining SR with software-defined perimeter (SDP), a new network security technology, is expected to solve the traditional problems such as data monitoring, denial of service, and new threats such as loop attack and label detection faced by SR data plane. Focusing on the security management of access devices in the SR data plane, first, this paper proposes an SR security model SbSR (SDP-based SR) based on SDP trust enhancement architecture, then, two-level SDP AH trust verification mechanism and 4 trust management mechanisms including initial trust value, trust evaluation, trust renewal, trust inheritance are designed. In the trust evaluation mechanism as the core of the model, System cloud grey model (1,1) weighted Markov prediction model is introduced to obtain real-time trust based on the historical behavior of device nodes, and 4 indexes, namely benign message ratio, loyal forwarding ratio, forwarding ratio stationarity coefficient, packet rate stationarity coefficient, are introduced to distinguish malicious devices from normal devices. Finally, the simulation test results of 5 security functions and security costs show that the proposed architecture can solve port scanning, traffic monitoring, topology detection, loop attack, and DoS attack of SR network data plane with an average access delay cost of 2.84 s for each new network agent, and realize multi-faceted protection of SR network data plane.

## Introduction

SR is the key technology of large-scale SDN (software-defined network) backbone network innovation at present. It takes source routing and statelessness as its key characteristics. Based on the labeling mechanism introduced by MPLS technology, it deletes the complicated LDP/RSVP-TE protocol required for label allocation, greatly simplifies the MPLS control plane and realizes an application-driven network^1–3^. Therefore, it is also known as "Next Generation MPLS", which has been deployed in top operators/OTT (Over the Top) networks at home and abroad, and can be implemented based on open source SDN routers such as OpenDaylight. SR only keeps the flow state in the message header, instead of the OpenFlow implementation in classical SDN which needs to keep the state for each hop of each flow and overcomes its convergence bottleneck^4–6^.

Essentially, SR adopts SDN architecture based on the transformation of the IP system, while it brings agility to future networks, in addition to the inherent threats of traditional IP networks such as traffic eavesdropping, service denial, and identity theft, SR also faces new hidden dangers such as single point failure of controller^7^ and service failure of SR nodes^8^ caused by the introduction of new components and new network layers. At present, the academic research on SR technology mostly focuses on function realization^9–12^, but there is little research on related security; to ensure its security, the industry is mainly based on hardware boundary security components such as firewall and VPN and lacks systematic security solutions. Because of the lack of deployment flexibility, this kind of border-based security component is hard to provide security protection for new application scenarios such as mobile internet, cloud computing, edge computing, IaaS, etc., and faces threats such as lateral movement and loopback attacks^13–16^. Therefore, it is urgent to provide a comprehensive security protection scheme for these new Zero-Trust application scenarios for SR.

SDP is a new general network defense scheme put forward by CSA(Cloud Security Alliance) in 2013 for the environment of ZTA(Zero Trust Network)^17^, which is essentially a kind of network defense scheme using SDP controller, application gateway (namely SDP Accept Host, SDP AH) and SDP Initial Host (SDP IH) and other software-defined logic components, and an enhanced access control architecture based on identity-based security boundary is built between network elements, which can realize the hiding of services and ports, and minimize the network attack surface. If SDP architecture is directly used to protect end-to-end services based on the SR network, it may be impossible to consider the protection of the network itself. It will also bring more security overhead. Therefore, this paper focuses on adapting the security architecture of SDP to the SR network and trust enhancement, and then combining it with the functional architecture of SR, to realize the benign coupling of security and functionality, to effectively deal with threats such as port scanning, denial of service and routing loop attack^18^.

Focusing on trust enhancement, this paper improves the traditional SDP architecture, mainly adding control plane information synchronization, SDP AH two-level verification, and 4 trust management mechanisms. Control plane information synchronization means that SDP controller is synchronized with SDN controller/SR PCE in SR network; SDP AH two-level authentication refers to the deployment of SDP AH components for SR domain entry nodes and service resources respectively, to realize the dual protection of the network itself and network resources; the 4 trust management mechanisms are trust initial value, trust evaluation, trust renewal, and trust inheritance mechanism, among which the most important one is trust evaluation mechanism. To obtain real-time trust, SCGM (1,1) weighted Markov prediction model suitable for random process state prediction is introduced, and its current trust is evaluated based on the historical performance of access nodes. To distinguish whether the node's historical performance is normal or not, 4 indexes, namely benign message ratio, loyal forwarding ratio, forwarding ratio stationarity coefficient, and packet rate stationarity coefficient, are introduced, which are used to characterize the label characteristics, forwarding characteristics, stability characteristics and rate characteristics of the messages sent by the nodes, respectively, to comprehensively judge malicious nodes. The function and security cost tests show that the proposed model is helpful to solve the common security threats of the SR data plane.

The contribution of this paper mainly lies in 3 aspects: First, three SR function models are summarized and put forward, and then the traditional and new security threats that its data plane faces are put forward; the second is to design an SDP trust enhancement architecture by improving the basic architecture of SDP for SR network and trust enhancement, and apply it to SR network for the first time, and realize a new SR data plane security model; thirdly, around the proposed security model, four types of trust management mechanisms, namely initial trust value, trust evaluation, trust renewal, and trust inheritance, and SPA(Single Packet Authorization) knock-on package that can prevent replay attacks are also proposed and implemented.

This paper is mainly composed of 6 sections, among which, in the second section, the SR functional model, SR native security mechanism, SR data plane security problems, existing SDN data plane security scheme and routing security scheme are summarized and proposed. The third section introduces the architecture design of the SbSR model and the communication methods of SR/non-SR components in this model. In “Mechanism and overhead of SbSR model” section introduces the access mechanism and trust enhancement management mechanism of the SbSR model and analyzes the main overhead of it. In “Simulation test and analysis” section, the security performance and overhead of the SbSR model are tested based on the EVE-NG simulation environment. In “Conclusion and future work” section summarizes the full text and looks forward to the next research.

## Related works

At present, there are few security models for protecting SR networks, and the academic circles have not started the research on the combination of SDP and SR. Therefore, in the related work, firstly, the functional models and native security mechanisms of SR are summarized. Then, the security problems of the SR data plane are put forward. Finally, the SDN data plane security scheme and main routing security mechanisms are introduced.

### SR function model

**Figure 1:**
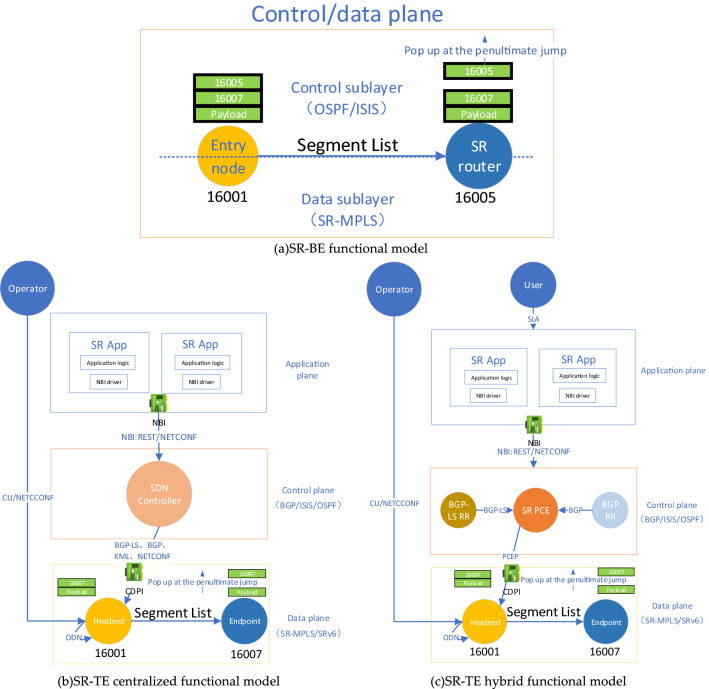
Typical SR function model.

SR can be divided into SR-BE (SR-Best Effort) for realizing equivalent shortest path forwarding in the domain and SR-TE (SR-Traffic Engineering) for realizing directional traffic engineering, and SR-TE can support centralized and hybrid deployment models^11^. Therefore, based on SR-BE/TE principle, this paper summarizes and refines the SR-BE, SR-TE centralized, and SR-TE hybrid functional models respectively^19,20^, as shown in Fig. 1a–c.

It can be seen that SR has innovated the typical three-layer functional model of SDN application plane, control plane, and data plane. Among them, in the SR-BE functional model shown in Fig. 1a, the traffic path is the optimal SR LSP calculated according to IGP shortest path algorithm, and the application plane is not especially emphasized. The functions of the control plane and data plane are integrated on the same router, which is separated from the SDN controller to realize the functions. In the SR-TE functional model shown in Fig. 1b and c, by coding and setting the source routing path information in the head-end nodes such as routers, hosts, devices, etc., the data stream is transmitted to the endpoint node through the path of the designated color mark according to the MPLS label stack, and 1 top label pops up every time it reaches the penultimate hop of the designated SID node, the source route can be configured by the operator through CLI, NETCONF, or explicitly specified by the controller through BGP, NETCONF, etc., or automatically configured by SR PCE (Path Calculate Element) through PCEP, or automatically generated according to the template by the head-end node itself through ODN (On-Demand Next-hop) function. The difference between the SR-TE centralized model and the hybrid model is that the former shown in Fig. 1b uses the centralized optimization of the SDN controller to control the network, while the latter shown in Fig. 1c comprehensively uses SR The centralized optimization of PCE and the distributed intelligence of components such as BGP RR (Router Reflector) and BGP-LS RR. The SDN controller used in the centralized model will not be repeated; the SR PCE used in the hybrid model is based on the NOS (Network Operating System) software used to abstract the entire network view on the designated router and is deployed as a network control functional component. SR PCE can provide a global network view for centralized applications in the application plane through the northbound interface through REST, NETCONF, and receives SR Policy, it can also initiate paths to data plane nodes through the southbound interface through PCEP, BGP, XML, and NETCONF. It is worth noting that the key automatic drainage and ODN functions in SR-TE reuse the automatic coloring mechanism of BGP and the distributed intelligence of nodes, and have nothing to do with the SDN controller^21,22^.

### SR native security mechanism

As a new implementation of SDN, literature^1^ pointed out that SR adopted a certain native security mechanism for security reasons, which is summarized into 5 categories in Table 1 in this paper. It can be seen that the protective effect of these endogenous safety mechanisms is relatively limited.

**Table 1:** SR native security mechanism.

Security mechanism	Implementation mode	Threats to guard against
Source routing	The head node encapsulates the label stack to specify the flow path	Routing tampering
Trust domain	The source routing information is only used within the boundary of the trust domain. When the data packet leaves the domain, the source route is cleared by setting the C-flag flag in SRH(SR Extended Header)	Label leakage
Package validation	RFC8754 stipulates that the optional TLV object field of SRH in the SRv6 message carries HMAC TLV	Message tamper
Load-balance	Anycast-SID will balance the traffic through a key single node to multiple nodes	Single point failure
Fault detection and recovery	Local trigger (BFD), remote intra-domain trigger (IGP flooding), remote cross-domain trigger (BGP-LS), end-to-end SR Policy survivability detection, explicit candidate path verification, dynamic candidate path recalculation, TI-LFA	–

### SR data plane security problems

At present, there is no systematic research on the security of the SR data plane. Since SR originates from SDN, we refer to SDN data plane security problems here. Literature^23^ divides attacks on the SDN data plane into 3 types: device attack, protocol attacks, and side-channel attacks, and other literature also analyze these 3 types of threats respectively. Among them, in the aspect of equipment attack, literature^24^ pointed out that OpenFlow switch flow table can be used to infer network parameters such as flow table capacity and flow table usage with high accuracy; in terms of protocol attacks, literature^25^ pointed out that malicious applications can install targeted flow rules by overwriting existing flow rules with matching fields that are not supported by hardware; in terms of side-channel attack, literature^26^ points out that input buffer can be analyzed to identify forwarding rules, and packet processing time can be analyzed to identify forwarding strategies.

**Figure 2:**
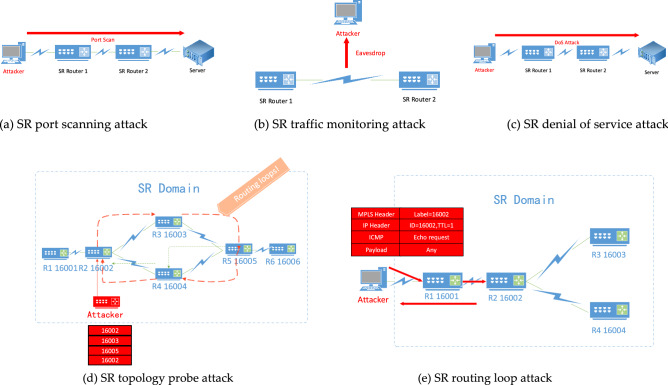
Common attack scenarios of SR (**a**) SR port scanning attack (**b**) SR traffic monitoring attack (**c**) SR denial of service attack (**d**) SR topology probe attack (**e**) SR routing loop attack

According to Fig. 1 and Table 1, SDN data plane security problems and experimental tests, data plane security problems that cannot be solved by SR native security mechanism can be divided into traditional problems and new problems, among which, traditional problems include port scanning, traffic monitoring, denial of service attack, side-channel attack, protocol attack based on IGP/BGP routing protocol vulnerabilities, etc., and new problems include device attack based on software and hardware vulnerabilities of SR router, topology detection based on label detection, loop attack based on a directional label^27^, etc. Figure 2 illustrates some typical attack patterns studied in this paper, among which, Fig. 2a illustrates that attackers scan the open ports of network service resources through the SR network; Fig. 2b shows that the attacker monitors the communication traffic between devices in the SR network. Figure 2c shows that an attacker makes a DoS attack on SR network service resources; Fig. 2d shows that the attacker probes SR network topology and node labels by constructing specific probe messages. Figure 2e shows that the attacker makes a loop attack on the SR network by constructing a routing loop attack message and maliciously occupies the network bandwidth.

### Existing SDN data plane security scheme and routing security scheme

In terms of existing SDN data plane security schemes, literature^28^ analyze challenges involved in protecting SDN data plane comprehensively and provide an in-depth look into available solutions and point their limitations. Literature^29^ presents a study and demonstration of some commonly seen internal security attacks and related countermeasures in programmable data planes using P4, a data-plane programming language, this study can provide users with the flexibility to add or drop security features in the deployed SDN switches. literature^30^ propose a new SDN-based data plane architecture called DPX (Data Plane Extended) that natively supports security services as a set of abstract security actions that are then translated to OpenFlow rule sets. DPX provides administrators a simplified method for configuring security services into the network. literature^31^ utilize OpenFlow possibilities and Machine Learning concept for the proposed Network Intrusion Detection System based on Deep Neural Network (NIDS-DNN). NIDS-DNN can extract network statistics from OpenFlow switches and process them with DNN. Then it will warn about attacks on the data plane and prevent malicious users from harming the network. Considering one nominal controller in charge of the data plane computation, literature^32^ designed a second one to control the consistency of the decisions made by the controller. Compared to related works, no direct exchanges between the controllers are required in this solution. In the aspect of routing security scheme, in literature^33^, probabilistic framework is proposed that facilitates data routing between the nodes and local cloud in an IoT network coupled with a multitier trust and encryption scheme for secure data delivery in the cloud-based IoT network. literature^34^ presents a security-aware routing mechanism and discusses with quality of service (QoS) factors such as throughput and accuracy for improving routing mechanism. In literature^35^, a multi-path QoS (quality of service) routing security algorithm based on blockchain by improving the traditional AODV (ad hoc on-demand distance vector) protocol is proposed, effectively in improving security and QoS. However, the above methods do not provide an overall protection solution from the perspective of zero-trust. The comparison between them and the scheme proposed in this paper is shown in Table 2.

**Table 2:** Comparison of 3 types of security solutions.

	General SDN data plane security solution	General routing security scheme	SbSR model
Means	P4, DPX, NIDS-DNN, more controllers, etc	Based on probability, based on security-aware routing, based on blockchain, etc	Based on SDP components
Advantages	Helps improve security and quality of service		Designed for segment routing; provides comprehensive protection; can be used in new zero-trust application scenarios
Disadvantages	Being not combined with the characteristics of source routing and segment labeling of SR; lack comprehensive protection; it is difficult to face new zero-trust application scenarios		Need to be improved for SR; it only supports the management of terminal devices, and cannot directly control routing devices

## Design of SbSR model

To build an SR security model based on SDP architecture, improve the security performance of SR data plane and reduce the security overhead, firstly, map SDP IH and network resources to SR data plane; then, an improved SDP trust enhancement architecture and SR data plane security model based on the former are proposed. Finally, the SPA package is designed.

### Data plane mapping of SDP IH and network resources

**Figure 3:**
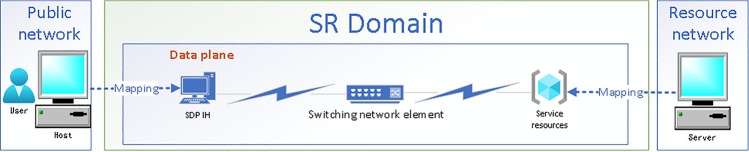
Data plane mapping of SDP IH and network resources.

Because SDP IH needs to be regarded as a network agent binding users and devices under the zero-trust architecture, and it should be deployed in the SR network data plane, not as an OpenFlow switch only responsible for forwarding and processing, so it needs to be modeled and mapped to a data plane network agent with only the functional attributes and user identities of the bottom 3 layers of OSI, that is, SDP IH is mapped to a network agent $N - agent$ running on the data plane through the data plane mapping function $\Omega$, as shown in formula (1) and (2). User ID *UID* is defined by formula (3), where $\Gamma$ is the attribute-identity mapping function, $ID_{user}$ is the user attribute set, $User_{N}$ are user names, $User_{C}$ are user categories, and $User_{{\text{P}}}$ are preset permissions for users; access objects such as network resources are mapped and modeled as data plane service resources similarly, as shown in Fig. 3.

1 $$ {\text{SDP IH}}_{{\text{i}}} \mathop = \limits^{def} \left\{ {\begin{array}{*{20}l} {{\text{UID + }}App_{i} + Format_{i} + Session_{i} } \hfill \\ { + Port_{i} + IP_{i} + MAC_{i} + Interface_{i} } \hfill \\ \end{array} } \right\} $$

2 $$ {\text{N - agent}} = \Omega ({\text{SDP IH}}_{{\text{i}}} )\mathop = \limits^{def} \left\{ {UID + IP_{i} + MAC_{i} + Interface_{i} } \right\} $$

3 $$ UID\mathop = \limits^{def} \Gamma (ID_{user} ) = \Gamma (User_{N} ,User_{C} ,User_{P} ) $$

### SbSR (SDP-based SR) model design

#### SDP trust enhancement architecture

In this paper, the proposed SR data plane security model based on SDP trust enhancement architecture is called SbSR. Compared with SDP basic architecture, the SDP trust enhancement architecture introduced in this paper mainly focuses on the key security factor of trust and adds the following three mechanisms.

Firstly, control plane information synchronization. In the SbSR model, there are two modes in the control plane: SDN controller + SDP controller and SR PCE + SDP controller, and mutual transport layer security (mTLS)^36^ connection is established to ensure information synchronization and control signaling transmission security. In both modes, the SDP controller is deployed in the direct communication channel of authentication traffic upload and control signaling to realize authentication evaluation of access hosts in the domain. The difference is that in the mode of "SDN controller + SDP controller", SDN controllers supporting SR such as OpenDaylight are used to control the SR router; in the mode of "SR PCE + SDP controller", physical or virtual routing nodes (such as Cisco IOS XR router) are used to enable the SR PCE function as SR PCE to control the SR router. SDN controllers and SR PCEs can be deployed in multiple units to achieve load balancing and disaster recovery.

**Figure 4:**
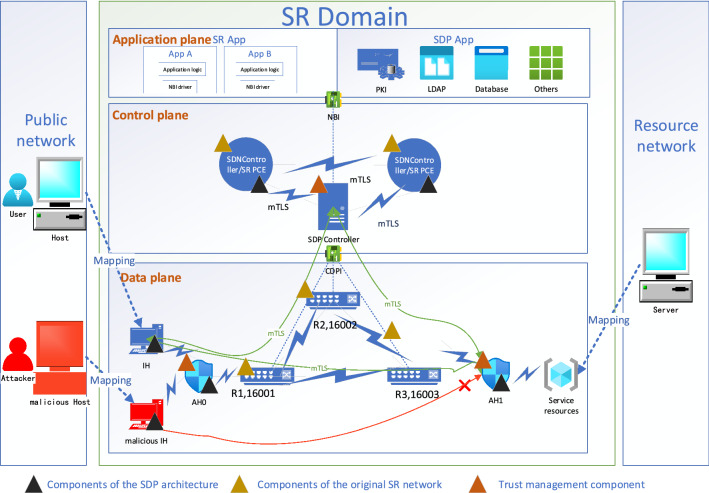
SbSR security model architecture.

Secondly, SDP AH two-level authentication. Two levels of SDP AH are deployed in the SbSR model, one is deployed between SDP IH and SR domain entrance router, and is called SDP AH_ent_, as shown in AH0 in Fig. 4, which is used to hide the network topology, that is, before SDP IH accesses SR domain router, it needs to request the SDP controller for authentication through SDP AH, and the classical SDP access process needs to be improved here, which is described in detail in “SR data plane security problems” section. The other one is deployed between the SR router and service resource as the Provider Edge (PE) node, which is called SDP AH_ser_, as shown in AH1 in Fig. 4, and is used to hide network services and prevent attackers from grasping the port information of service resource. Two-level access control mechanisms are set for SDP IH corresponding to the two SDP AH deployment positions, that is, SDP IH is allowed to access the router after its trust value reaches the SR domain access threshold, and can only access the service resource after the trust value reaches the designated service resource trust threshold. The reason why the two-level access control mechanism is set is that different service resources have different trust threshold requirements. Imagine that if the SDP AH_ser_ for service resources is canceled and only the SDP AH_ent_ at the domain entrance is kept, if the SDP AH deployed at the domain entrance sets a unified trust threshold for service resources at this time, the fine-grained control of SDP IH's access to various remote service resources cannot be realized. If SDP AH_ent_ manages and controls all kinds of remote service resources in a classified and integrated way, it will increase the access delay of normal SDP IH too much, and when adding new service resources, it is necessary to change the integrated SDP AH_ent_ to reduce the network scalability. If SDP AH_ent_ is canceled, it will not be possible to verify the behavior of hosts outside the domain after accessing the network.

Thirdly, the trust management mechanism. It will be introduced in detail in “SbSR trust enhancement management mechanism” section after introducing the SbSR model architecture and access mechanism.

#### SbSR model architecture

Figure 4 shows the basic architecture of the SbSR security model, in which the application plane consists of SR applications and SDP applications such as PKI (Public Key Infrastructure), LDAP (Lightweight Directory Access Protocol), and device authentication. The control plane consists of an SDP controller and SDN controller/SR PCE. In the data plane, users, hosts of the public network, and server groups of the resource network are mapped to SDP IH and service resources respectively through the data plane mapping function $\Omega$, in which some SDP IH may be malicious, and the data plane switching equipment is SR router, which is assigned SID in advance. To ensure the security of the control plane, SDP/SDN controller and SR PCE only connect routers and applications in the SR domain and do not connect any network elements outside the domain, thus reducing the network attack surface to SDP IH. Based on this attack surface condition and focusing on data-plane security, the original control plane components and SR router of the SR network are trusted by default.

#### Communication between SR and non-SR components in the SbSR model

To ensure that SR components such as SR PCE/SDN controller and SR router in SR domain communicate with non-SR components such as SDP IH and SDP AH normally, the following control plane and data plane SR/ non-SR component communication mechanisms are designed.

##### Control plane

The MPLS architecture supports the coexistence of non-SR control plane protocols such as LDP, RSVP-TE, and SR control plane protocols such as OSPF, ISIS, and BGP. Therefore, by running MPLS Label Manager (LM) components in the MPLS control plane of each node, local labels distributed by different label distribution protocols will not conflict.

##### Data plane

The LM component of each node guarantees the unique distribution of local labels so that LSP (Label Switching Path) generated by different MPLS control plane protocols can coexist. Therefore, the communication between SR components and non-SR components mainly involves two scenarios: one is MPLS to IP, such as SR router transmits data messages to SDP IH/SDP AH that does not support MPLS; second, IP to MPLS, such as SDP IH/SDP AH which does not support MPLS, transmits data and authentication packets to SR router.

**Figure 5:**
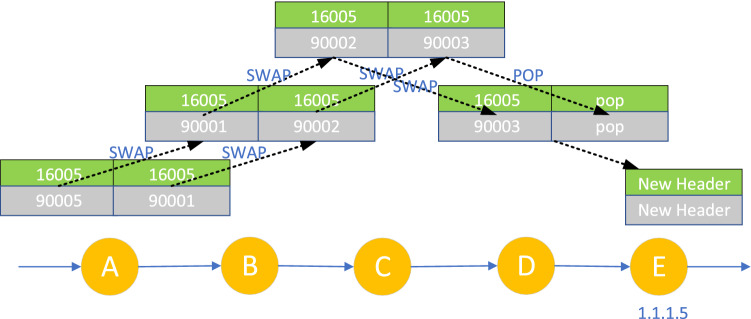
MPLS-to-IP communication scenario.

In the MPLS-to-IP communication scenario, the traffic is sent out by SR routing equipment and the inbound label is not empty. Because the LM component management of each node ensures that the local label is unique, the forwarding entries from MPLS to MPLS/IP are in one-to-one correspondence with the local/inbound labels, these MPLS forwarding entries can coexist. As shown in Fig. 5, nodes A, B, C, D, and E all enable SR and LDP, among which, Nodes A, B, C, and D adopt the default SRGB [16000-23999]. Node E announces its loopback address prefix 1.1.1.5/32 and its corresponding Prefix-SID index 5, and requests the default penultimate pop-up operation for this Prefix-SID. At this time, the local label assigned by nodes A, B, C, and D to the Prefix-SID of node E is 16,005 SRGB starting value 16,000+ index value 5), the LDP label is assigned and announced for the loopback address prefix of node E, and the LDP local label assigned by node A to the loopback address prefix of node E is 90,005(LDP label range starting value 90,000+ index value 5). In the label stack of each node in Fig. 5, the left side is the inbound/local label and the right side is the outbound label. When the data packet with the top label of 16,005 arrives at node A, it will be forwarded to nodes B and C in turn according to the green label entry in the figure and the SR penultimate hop operation with the type of "switching". At node D, the top label is popped up, and at node E, the packet is processed based on the new header exposed after popping up the top label. When the packet top-labeled 90,005 enters node A, the operation is similar.

**Figure 6:**
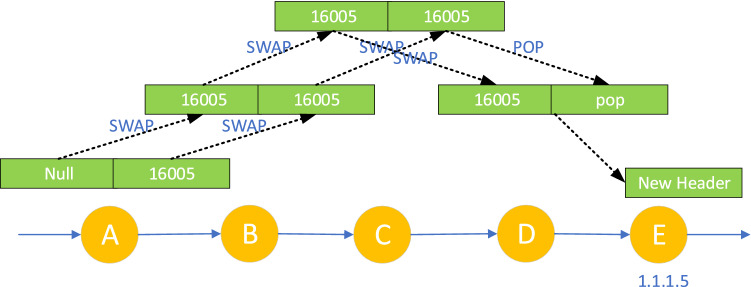
IP-to-MPLS communication scenario.

In the IP-to-MPLS communication scenario, for example, SDP IH requests the SDP controller to verify its identity, when the IP traffic with an empty inbound label reaches the SR domain entry node, it is divided into FEC (Forwarding Equivalence Class) by matching the longest prefix of the destination address, and then SR label is pressed into the packet according to FEC, as shown in Fig. 6, all nodes are enabled with SR, and the label configuration is the same as that in Fig. 5. When the unlabeled IP data packet arrives at node A, it is pushed into SR label 16,005, and forwarded to nodes B and C in turn according to the green label entry, with the penultimate hop operation of type "Exchange", the top label is popped up at node D, and the data packet is processed at node E based on the new packet header exposed after popping up the top label.

## Mechanism and overhead of SbSR model

### SbSR access mechanism

The access mechanism of SbSR is: to implement trust management for SDP IH, which is the possible attack surface of the network, that is, providing the east–west access control mechanism of the data plane to prevent the malicious SDP IH from moving horizontally after accessing the network, specifically according to the following access process.

*Step 1* Assign the SRGB label to the routers in the SR domain and enable SR service.

*Step 2* SDP controller goes online, connects to external authentication and authorization SDP application, and establishes mTLS connection with SDN controller/SR PCE in the domain; meanwhile, mTLS connection is also established between SDN controller/SR PCE.

*Step 3* Two levels of SDP AH are connected in series to the domain entrance node and service resources respectively. By default, the "drop-all" access strategy is adopted, and all visiting data packets are discarded (but the SPA knocking data packets are recognized, and only those that pass the verification are responded to). SDP AH transmits its SPA packets to the SDP controller through the directly connected routing device to request its authentication identity. If the authentication passes, the mTLS connection is established between the SDP controller and SDP AH through the routing device.

*Step 4* Before an SDP IH accesses the SR network, it accesses the network agent authentication component of the application plane in advance, and the latter reads its security information items and enters them into the network agent security information database.

*Step 5* SDP IH connects to the routing device through SDP AH_ent_, and sends the SPA authentication package containing its $N - agent$ information to SDP AH_ent_. SDP AH_ent_ does not respond, and forwards it directly to the SDP controller through the domain entrance routing node, requesting its authentication identity and issuing access credentials.

*Step 6* The SDP controller calls the application plane identity authentication authorization component to authenticate the SDP IH identity. If it passes, it authorizes the accessible SDP AH list, temporary access credentials, and policies, but does not send them for the time being.

*Step 7* SDP controller informs all SDP AHs involved in authorization list of authorized SDP IH identity, temporary access credentials, and policies through mTLS link.

*Step 8* SDP AH_ent_ modifies its own packet filtering rules and informs SDP IH of its accessible SDP AH list, temporary access credentials, and policies.

*Step 9* SDP IH uses the access credentials of SDP AH_ent_ and its SPA package to establish mTLS connection with SDP AH_ent_ and obtain the access right of SR domain data plane; if SDP IH needs to access SDP AH_ser_, it takes the domain entry node as the head node, generates a Segment list to the SDP AH_ser_ directly connected routing device, and presses into the packet header, taking SPA containing access credentials as the packet load, requests access to the specific SDP AH_ser_ through the SR source routing transmission mechanism and establishes mTLS connection for it through the intermediate router after verification. At this time, the normal SDP IH will successfully access the service resources protected by SDP AH because the authentication authorization is passed, as shown by the green line in Fig. 4; Malicious IH will fail to authenticate and cannot be accessed because it cannot obtain access credentials, as shown by the red line in Fig. 4.

*Step 10* SDP AH_ent_, which is directly connected by SDP IH, conducts continuous trust evaluation on its behavior during the visit. If its trust value is higher than its trust threshold when SDP IH temporary access credentials expire, it is allowed to renew its credit lease and postpone its credentials; otherwise, close the corresponding mTLS connection, and the connection can only be restored after re-verification by SDP controller.

### SbSR trust enhancement management mechanism

As the basis of the SbSR model, the SDP trust enhancement architecture is essentially a zero-trust architecture, the core of which is trust enhancement management. To fully consider trust, some common attack styles are defined first.

**Definition 1** Switch attack.

The attack in which malicious nodes sometimes take benign or malicious actions to confuse security checks.

**Definition 2** Masquerade attack.

The attack in which malicious nodes hides their malicious behavior according to the detection rules during verification, and executes the malicious behavior after it passes the verification.

**Definition 3** Unknown attack.

The attack which pattern is unknown and the existing verification rules cannot match.

#### User ID code, device ID code, and SDP AH trust threshold

The SDP controller evaluates the trust of SDP IH, mainly considering trust value its corresponding $N - agent$, including user trust and device trust. We set two-level ID codes for users and devices according to different granularity, and defining the primary ID code of user *i* as shown in formula (4), which is used to encrypt and identify different user IDs in the SR domain; as shown in formula (5), the secondary ID code is used to identify the instantaneous identities of different users, $timestamp$ is the time stamp and accurate to seconds, it is used to provide time factors for user identities; the primary ID code of the device j is shown in formula (6), which is used to encrypt and identify different devices in SR domain; the secondary ID code is shown in formula (7), which is used to identify different interfaces of different devices. In formula (4) and (5), $Key_{seed}$ is preset by the administrator in the authentication component in the application plane, and can't be read. It can only be verified by the "challenge-response" mechanism, and users can use it by dedicated authentication accessory hardware or by human memory (in fact, once $Key_{seed}$ leaked, it will not fundamentally endanger the security of the SR domain. $ID_{i}$ is the unique ID of user i, such as the 18-digit ID number, and the identity of user should be verified by biometrics in a qualified verification environment; $IP_{j}$,$MAC_{j}$,$Interface_{j}^{k}$ is the IP address of device j for access, the MAC address of the corresponding network card and the k interface respectively.

4 $$ User_{i}^{ID1} = HMAC\left( {Key_{seed} + ID_{i} } \right) $$

5 $$ User_{i}^{ID2} = HMAC\left( {Key_{seed} + ID_{i} + timestamp} \right) $$

6 $$ Device_{j}^{ID1} = HMAC\left( {IP_{j} + MAC_{j} } \right) $$

7 $$ Device_{{j_{k} }}^{ID2} = HMAC\left( {IP_{j} + MAC_{j} + Interface_{j}^{k} } \right) $$

At the same time, set the trust threshold for all SDP AHs in the domain, and set the trust threshold of SDP AH m as ${\text{Th}}_{m}$.

#### Trust initial value, trust evaluation, trust renewal, and trust inheritance mechanism

In the zero trust architecture, no subjective default trust is granted to any user or device. To improve its service performance and reduce the workload of trust granting based on ensuring the security of SbSR architecture, it is necessary to balance the granularity of trust management and trust management overhead. Therefore, referring to the historical performance of network agents, 4 mechanisms of initial trust value, trust evaluation, trust renewal, and trust inheritance are set up. Among them, the initial trust mechanism is used to grant initial trust to the network agent for the first time; trust evaluation mechanism, which is used for obtaining current trust by real-time evaluation of the behavior of the network agent after accessing the network; trust renewal mechanism, which is used to allow normal network agent renewal trust after accessing the network, and to extend the use period of access credentials; trust inheritance mechanism, which is used to allow the new network agent associated with the existing network agent to inherit part of its trust value when entering the network.

##### Trust initial value mechanism

To cancel the default trust, when initial trust is granted to users and devices that do not record the corresponding primary ID code in the authentication component, the default trust value of 0 is granted to them. When granting initial trust to new network agents, on the one hand, multi-factor authentication information such as firewall version and port opening of the network agent's own device is difficult to be transmitted through a single SPA package; on the other hand, malicious network agents may intentionally upload "perfect information" or cheat SDP controller through replay attacks, so before a network agent accesses SR network, it is forced to access the network agent authentication component of the application plane in advance, and the latter reads its security information items and enters them into the network agent security information database. Table 3 lists some items of network agents' security information.

**Table 3:** Part of network agent security information items.

Object category	Primary index	Secondary index	Three-level index
User	No history of malicious behavior	Save private key with trusted platform module (TPM)	Firewall versions with known vulnerabilities are not enabled
		Users belong to users inside the network	A user forgot his password
		User category (administrator, front desk operator, background operator)	A user requests a service that is higher than his role authority
Equipment		Manufacturers and suppliers are trusted manufacturers list	The system authentication hardware token is missing

When the network agent requests the SDP controller to verify the identity, the SDP controller uses the primary ID code of the user and device contained in its SPA package to match in the network agent security information base and calls the external information source to check whether there is any malicious behavior record of the corresponding user or device. There are 2 ways to grant initial trust here:

Method 1: Directly grant the domain minimum access right to the nodes with no malicious history and complete identity information after investigation, that is, grant them the access certificate set $CertA$ of SDP AH list $AH_{A}$ that meets the trust threshold and the validity period $t_{{{\text{CERT}}_{i}^{IH0} }}$ of the certificate $Cert_{{\text{i}}}^{IH0}$, where $AH_{A}$ and $CertA$ are shown in formulas (8) and (9) respectively; then, evaluate their trust based on their real-time behavior. Those who have a record of malicious acts will not be authorized. This method can realize fast authorization, but its disadvantage is that it requires high real-time and security of trust evaluation algorithm.

8 $$ AH_{A} = \left\{ {AH_{ent} ,AH_{ser1} ,AH_{ser2} ,AH_{ser3} , \ldots ,AH_{sern} } \right\} $$

9 $$ CertA = \left\{ {Cert_{0}^{IH0} ,Cert_{1}^{IH0} ,Cert_{2}^{IH0} ,Cert_{3}^{IH0} , \ldots ,Cert_{m}^{IH0} } \right\} $$

Method 2: SDP controller uses its trust calculation engine to grant the 3-level evaluation coefficient $\alpha ,\beta ,\gamma$ satisfying formula (10) to the first, second, and third-level indexes of network agent security information items, then check and calculate network agent’s trust value item by item based on all security information items, and grants initial trust to nodes satisfying formula (11) if there is no malicious behavior record, but this method cannot cope with the masquerade attack and unknown attack.

10 $$ \partial \ge \beta \ge \gamma $$

11 $$ W_{{N - agent_{i} }} \ge Th_{{SDP{\text{AH}}_{ent} }} $$

In this paper, method 1 is adopted to grant initial trust, that is, after examination, the initial trust value of ${\text{Th}}_{{SDPAH_{ent} }}$ is directly granted to the node, so that it can just access the router in the domain. This is because "looking forward" unknown attacks are difficult to avoid fundamentally, while "looking backward" evaluation of historical behavior of nodes is relatively easy to realize.

##### Trust evaluation mechanism

The access node's trust history behavior is evaluated in real-time, and the normal behavior is defined as packets sent by SDP IH are not SR label probe packets or SR label loop packets, and the received messages are faithfully forwarded^37^. Therefore, 4 indexes, benign message ratio, loyal forwarding ratio, packet rate stationarity coefficient, and forwarding ratio stationarity coefficient, are introduced^38^.

**Index 1** Benign message ratio.

The proportion of non-SR label detection packets or label loop packets in the messages sent by the node.

**Index 2** Loyalty forwarding ratio.

The proportion of the number of messages actually forwarded by a node in the total number of messages that should be forwarded.

**Index 3** Forwarding ratio stability coefficient.

The stability of the node loyalty forwarding ratio sequence (measured according to the gray prediction model).

**Index 4** Packet rate smoothness coefficient.

The smoothness of the flow rate sequence sent by the node to the SR network (measured according to the gray prediction model).

First of all, to consider index 1, SDP AH needs to check whether the MPLS header label in the received packet has a quantitative relationship such as increasing and decreasing, and then judge whether it is an SR label probe packet because the label probe packet is usually based on hop-by-hop detection of the MPLS label increasing for unknown labels, that is, there must be a certain amount of relationship in the MPLS header label of the packet detection packet.

In addition, SDP AH needs to judge whether there is a label loop in the received MPLS header. Because the judgment of label loop cannot be based solely on whether there are duplicate labels in the MPLS label stack, it needs to be combined with SR topology information, which is beyond the capability range of SDP AH. Here, the negative feedback of bandwidth decrease is used to consider whether there is a routing looping attack packet, that is, when the available bandwidth of SR network decreases, it is judged whether there is a looping packet, and then it is judged whether the node sends a routing loop attack packet based on the traceability of the source IP in the loop packet. The node with index 1 lower than 1 is punished by trust, and if it continues to send SR label probe packets or routing loop packets, its access connection will be blocked.

Secondly, to consider indexes 2 and 3, the communication period from the establishment of mTLS connection to the evaluation time is regarded as the evaluation interval T, which is divided into t segment evaluation intervals. It is considered that normal SDP IH tends to faithfully forward data normally with a high probability (the probability is not less than 0.5), while malicious SDP IH is unfaithful, that is, it forwards data normally with a low probability (the probability is less than 0.5). If the number of messages that SDP IH should forward in the j-th segment evaluation interval is *f*_1_, and the number of packets actually forwarded is *f*_*2*_, the loyalty forwarding ratio of the SDP IH in this evaluation interval is calculated as shown in formula (12)^37^.

12 $$ F_{AH,IH}^{j} = \frac{{f_{2} }}{{f_{1} }} $$

Based on maintaining normal forwarding, the SDP IH node carrying out a DoS attack maliciously increases the transmission traffic to SDP AH, at this time $F_{AH,IH}^{j}$ can't be used as a valid index for judging the malicious behavior of the node. Therefore, it is necessary to consider index 4, that is, measure the stationarity of SDP IH to SDP AH outbound forwarding traffic rate sequence $V_{AH,IH}$ within the evaluation interval of the t segment. According to the grey prediction model, the defined ratio sequence $\varepsilon$ and the packet rate stationary coefficient $\mu$ are shown in formulas (13) and (14) respectively, and those $\varepsilon_{i}$ satisfying formula (15) are regarded as fluctuation values^37^.

13 $$ \varepsilon = \left\{ {\varepsilon_{1} ,\varepsilon_{2} ,\varepsilon_{3} , \ldots ,\varepsilon_{t - 1} } \right\},\varepsilon_{i} = \frac{{V_{AH,IH}^{i + 1} }}{{V_{AH,IH}^{i} }} $$

14 $$ \mu = \frac{{\sum\nolimits_{i = 1}^{t - 1} {\left| {\varepsilon_{k} - \min (\varepsilon )} \right|} }}{t - 1} $$

15 $$ \left| {\max (\varepsilon_{i} ) - \min (\varepsilon_{i} )} \right| > \mu $$

To distinguish between normal high-traffic data transmission and malicious denial of service attacks, the bandwidth of the service resources Server and SDP controller with the lowest service performance (measured by bandwidth) is set as $\min (Server|SDP_{C} )$ here. Once the expression (16) is satisfied, it is treated as a DoS attack and the network connection of this SDP IH is immediately cut off, its access credentials are cleared, and its behavior is recorded in the network agent security information base.

16 $$ \max (\varepsilon_{t} ) \times V_{AH,IH}^{t} \times \frac{t}{T} > 1.8\min (Server|SDP_{C} ) $$

If the number of interactive messages between nodes is too small in the evaluation interval T, and the calculation of the loyalty forwarding ratio of SDP IH will produce big errors, therefore, an interactive function $\delta (n)$ is introduced. In the function design, arctan(n) which monotonically increases with the increase of the number of interactive messages n and gradually approaches $\frac{\pi }{2}$ is optimized as shown in formula (17)^37^, so that it approaches 1 with the increase of n, which can be used for adjusting $F_{AH,IH}^{j}$.

17 $$ \delta (n) = \frac{\arctan (n)}{\pi } + \frac{1}{2} $$

After adjustment, when n is not 0, $F_{AH,IH}^{j}$ is shown in formula (18), where $F_{0}$ is the default forwarding ratio of SDP IH, that is, the initial message forwarding ratio when there is no interaction with SDP AH, and when n is 0, $F_{AH,IH}^{j}$ is shown in formula (19).

18 $$ F_{AH,IH}^{j} = \frac{{f_{2} }}{{f_{1} }}\delta (n) + F_{0} (1 - \delta (n)) $$

19 $$ F_{AH,IH}^{j} = F_{0} ,F_{0} \in (0,1) $$

At this time, the SDP IH loyalty forwarding ratio sequence $F$ corresponding to the available t segment evaluation interval can be calculated, and it can be used to predict $F_{AH,IH}^{t + 1}$. On the premise that formula (16) is not satisfied, $F_{AH,IH}^{t + 1}$ will be regarded as the trust value currently evaluated by SDP AH based on SDP IH historical performance, that is, the trust value given by the node is equal to its loyalty forwarding degree. The sequence $F$ and the ratio sequence $\tau$ derived from $F$ is shown in formula (20) and (21).

20 $$ F = \left\{ {F_{AH,IH}^{1} ,F_{AH,IH}^{2} ,F_{AH,IH}^{3} , \ldots ,F_{AH,IH}^{t} } \right\} $$

21 $$ \tau = \left\{ {\tau_{1} ,\tau_{2} ,\tau_{3} , \ldots ,\tau_{t - 1} } \right\},\tau_{l} = \frac{{F_{AH,IH}^{l + 1} }}{{F_{AH,IH}^{l} }} $$

Considering that malicious nodes may use switch attacks to mislead the prediction based on SDP IH historical behavior, it is judged whether there is a switch attack based on the sequence *F*. If there is a switch attack, the sequence *F* will have multiple overall migrations with each fluctuation. Therefore, the fluctuations in the sequence are divided into accidental fluctuations and migration fluctuations, in which the sequence will quickly return to normal after accidental fluctuations, while migration fluctuations will lead to overall migration of numerical distribution of the sequence. To separate the fluctuation value from the sequence $F$, according to the grey prediction model, similar to formula (14), the forwarding ratio stationarity coefficient $\theta$ is defined as shown in formula (22) based on the ratio sequence $\tau$.

22 $$ \theta = \frac{{\sum\nolimits_{l = 1}^{t - 1} {\left| {\tau_{l} - \min (\tau )} \right|} }}{t - 1} $$

The value $\tau_{l}$ satisfying formula (23) is regarded as the fluctuation value.

23 $$ \left| {\max (\tau_{l} ) - \min (\tau_{l} )} \right| > \theta $$

The sequence consisting of all the fluctuation values is regarded as a fluctuation sequence $W$, and the sequence $\tau$ from which the fluctuation values are removed is recorded as $\tau ^{\prime}$. To measure the increment caused by fluctuation of the series, the variable $\lambda_{t}$ of t is introduced to satisfy the expression (24).

24 $$ \left\{ {\begin{array}{*{20}l} {\lambda_{t} = 1,} \hfill & {\quad \tau_{l} > \tau_{l - 1} } \hfill \\ {\lambda_{t} = - 1,} \hfill & {\quad \tau_{l} \le \tau_{l - 1} } \hfill \\ \end{array} } \right.; $$

If the fluctuation values in the fluctuation sequence $W$ are all migration fluctuations, and 1 and − 1 appear alternately in the sequence $\lambda_{t}$, then SDP IH is likely to implement a switch attack. At this time, it is determined that it is a malicious node, and judge it is not trusted and entered its behavior into the network agent security information base.

After eliminating the possibility of switch attack, $F_{AH,IH}^{t + 1}$ will be predicted based on the sequence $F$, SCGM (1,1) weighted Markov prediction model is specifically adopted. At this time, according to whether $F_{AH,IH}^{t}$ is accidental fluctuation in the nearest evaluation interval from $F_{AH,IH}^{t + 1}$, the following calculation of $F_{AH,IH}^{t + 1}$ is made^37^:

(1) If $F_{AH,IH}^{t}$ is an accidental fluctuation, $F_{AH,IH}^{t + 1}$ is quite different from $F_{AH,IH}^{t}$, but close to the non-fluctuation value in the series, so it is directly predicted by SCGM (1,1) weighted Markov prediction model, as shown in formula (25).

25 $$ F_{AH,IH}^{t + 1} = SW{\text{MPred(F)}} $$

(2) If $F_{AH,IH}^{t}$ is non-fluctuation or migration fluctuation, $F_{AH,IH}^{t + 1}$ is close to $F_{AH,IH}^{t}$ at this time, and $F_{AH,IH}^{t + 1}$ is predicted by the above prediction model and $F_{AH,IH}^{t}$, as shown in formula (26).

26 $$ F_{AH,IH}^{t + 1} = SW{\text{MPred(}}\tau ^{\prime}{)} \times F_{AH,IH}^{t} $$

Through the above evaluation process, the real-time trust of SDP IH can be obtained, as shown in formula (27).

27 $$ TV_{{IH_{i} }} = F_{AH,IH}^{t + 1} $$

##### Trust renewal mechanism

When the SDP IH access credentials expire, if its real-time trust value is higher than the trust threshold of a certain SDP AH, it is allowed to renew the trust credentials equivalent to that SDP AH. At this time, the trust value of SDP IH $TV_{{IH_{i} }} (l)$ is a variable of the number of renewal rounds *l*. To make its trust increase with the number of rounds l, the trust gain brought by each renewal round decreases, the trust gain brought by renewal is not more than 1/2, and the total trust value is not more than 1, as shown in formula (28), there are two types of commonly used growth convex functions that meet the conditions, y1, and y2. Here we choose the exponential function and design the trust value $TV_{{IH_{i} }} (l)$ growth mechanism as shown in formulas (29) and (30).

28 $$ \left\{ {\begin{array}{*{20}l} {y1 = \frac{1}{2} - \frac{1}{{2e^{l} }}} \hfill \\ {y2 = \frac{\arctan l}{\pi }} \hfill \\ \end{array} } \right. $$

29 $$ TV_{{IH_{i} }} (l) = TV_{{IH_{i} }} (l - 1) + \frac{1}{2} - \frac{1}{{2e^{l} }} $$

30 $$ \left\{ {\begin{array}{*{20}l} {TV_{{IH_{i} }} (l) \in (0,1)} \hfill \\ {l \ge 1,\;l \in {\rm Z}} \hfill \\ \end{array} } \right. $$

At the same time, to prevent the unknown threats that the above 4 indexes failed to detect, SDP IH is not allowed to renew the lease permanently. Therefore, from the first round of renewal, an attenuation factor $p$ that increases with the network access time t is introduced for SDP IH’s trust value, which only acts on the historical trust in the last round of renewal of trust. If the temporary access credentials are valid for k, the trust attenuation function is set as shown in formula (31).

31 $$ \Delta (l) = e^{ - pt} = e^{ - pkl} $$

Finally, punish the bad operations of SDP IH that are not measured by indexes 1–4, such as logging in with wrong access credentials, and the negative feedback accumulates on its trust value. To ensure that the first negative feedback is low (because the trust degree is the maximum of 1, the first negative feedback is set to be less than 0.1 here), and gradually increase with the increase of negative feedback, by modifying the function y1 based on the initial requirements, the trust penalty function is set as shown in formula (32), in which *m* is the number of bad operations.

32 $$ P(m) = 0.4 - \frac{1}{{e^{m} }} $$

The attenuation factor and penalty factor are added to formula (29), and the correction is shown in formulas (33) and (34).

33 $$ \begin{aligned} TV_{{IH_{i} }} (l) & = TV_{{IH_{i} }} (l - 1) \times \Delta ({\text{l}}) + \left( {\frac{1}{2} - \frac{1}{{2e^{l} }}} \right) - \left( {0.4 - \frac{1}{{e^{m} }}} \right) \\ & = TV_{{IH_{i} }} (l - 1) \times \Delta ({\text{l}}) + \frac{1}{{e^{m} }} + \frac{1}{{2e^{l} }} + 0.1 \\ \end{aligned} $$

34 $$ \left\{ {\begin{array}{*{20}l} {TV_{{IH_{i} }} (l) \in (0,1)} \hfill \\ {l \ge 1,\;\;l \in {\rm Z}} \hfill \\ {m \ge 1,\;\;m \in {\rm Z}} \hfill \\ \end{array} } \right. $$

If the trust of an SDP IH is degraded due to excessive malicious behavior, the user is suspended from using his $Key_{seed}$ to prevent his subsequent application for authentication; at the same time, the malicious packets such as loop packets and probe packets in the network will be cleared.

##### Trust inheritance mechanism

To realize a "quick start", speed up the access of network agents to service resources, and set up a trust inheritance mechanism. The user who has recorded the primary ID code and no malicious behavior record in the network agent is regarded as an "old user". After the initial trust is granted to the user, refer to the trust value of the network agent corresponding to the secondary ID code of the user in the recent time interval, and multiply it by a unified user confidence factor $\nu$ to give the network agent a trust value increment, as shown in formula (35). The user confidence factor $\nu$ is determined by the trust performance of all users in the network in recent time, which is used to ensure that the network agent can reduce verification moderately, as shown in formula (36), where $\Lambda$ is the user confidence factor determining function.

35 $$ TV_{{User_{i}^{ID1} (t)}} = Th_{{SDPAH_{ent} }} + \nu TV_{{User_{i}^{ID2} (t - 1)}} $$

36 $$ \nu = \Lambda \left( {TV_{{User_{1}^{ID1} (t - 1)}} ,TV_{{User_{2}^{ID1} (t - 1)}} , \ldots ,TV_{{User_{n}^{ID1} (t - 1)}} } \right) $$

The device with the primary ID code recorded but the secondary ID code not recorded in the network agent is regarded as the "old device" with the "new port". After the initial trust is granted to it, the trust value of the network agent is incremented by referring to the minimum trust value of each port where the device is connected to the network and multiplying by the uniform device confidence factor $\sigma$, as shown in formula (37). $\sigma$ is determined by the trust performance of all access devices in the network in the recent period, as shown in formula (38), where $\Psi$ is the device confidence factor determining function.

37 $$ TV_{{Device_{j}^{ID1} (t)}} = Th_{{SDPAH_{ent} }} + \sigma \times \min \left( {TV_{{Device_{{j_{k} }}^{ID2} (t - 1)}} } \right) $$

38 $$ \sigma = \Psi \left( {TV_{{Device_{1}^{ID1} (t - 1)}} ,TV_{{Device_{2}^{ID1} (t - 1)}} , \ldots ,TV_{{Device_{m}^{ID1} (t - 1)}} } \right) $$

If the secondary ID code of the device in the network agent has been recorded, the device is regarded as the "old device" using the "old port". After the initial trust value is granted to the device, refer to the trust value of the device when it used the port to maintain a normal connection in the network last time, multiply it by the device confidence factor $\sigma$, and give an increment to the trust value of the network agent, as shown in formula (39).

39 $$ TV_{{Device_{j}^{ID1} (t)}} = Th_{{SDPAH_{ent} }} + \sigma \times TV_{{Device_{{j_{k} }}^{ID2} (t - 1)}} $$

#### SPA package settings

**Figure 7:**
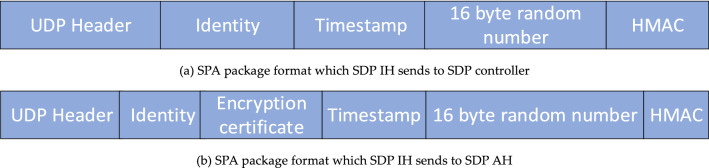
SPA package format.

To reduce the network attack surface, the SPA package adopts connectionless UDP package^39^ by default, as shown in Fig. 7a and b respectively, that is, when SDP IH requests the SDP controller to verify its own identity, the SPA packet load includes its 32-byte identity information (16-byte primary ID code of user and 16-byte primary ID code of device), timestamp used to prevent replay attacks, 16-byte random data, and message digest HAMC; after the SDP controller issues the private key used to verify the access credentials of the SDP IH to the SDP AH, and then issues the access credentials carried by the certificate and the public key of the SDP AH to the SDP IH, then the SDP IH requests the SDP AH to verify with a SPA package, its payload is an encrypted certificate, identity information, timestamp, 16-byte random data and corresponding HMAC encrypted with a public key. SDP AH uses its private key to decrypt and verify the validity of the certificate. The HMAC attached to the two kinds of SPA packages are shown in formulas (40) and (41) respectively.

40 $$ HMAC_{SPA - id} = HMAC\left( {User_{i}^{ID1} ,Device_{j}^{ID2} ,tstamp,data} \right) $$

41 $$ HMAC_{SPA - cert} = HAMC\left( {Cert_{m}^{{IH_{i} }} ,User_{i}^{ID1} ,Device_{j}^{ID2} ,tstamp,data} \right) $$

#### SbSR overhead analysis

The security overhead of the SDP access control mechanism introduced by SbSR model can be divided into four parts: component activation, access control, credential verification, and component synchronization, which are measured by time delay.

#### Component activation overhead

Refers to the time taken by all SDP components from activation to service start after accessing the SR domain, and it is generated at once for all. Referring to the open-source SDP implementation example, this process takes seconds.

#### Access control overhead

Refers to the cost of access control for network agents accessing the network, which is divided into four aspects: initial trust evaluation, trust evaluation, trust renewal, and inheritance evaluation. Among them, in the initial evaluation of trust, it is necessary to conduct 16-byte value matching according to the primary ID code of user and device in malicious behavior records of users and devices at the same time respectively. If the number of users accessing the network is n and the number of devices is m, the trust engine can calculate the number of 16-byte records to be matched every millisecond as *k*, because there is formula (42) in the network, the delay cost is much less than 1 ms, which can be ignored. When evaluating trust, the trust engine component set in SDP AH_ent_ is used to evaluate at every evaluation interval t (set less than 1 s), and the evaluation is synchronized with network forwarding, since the evaluation time is less than t, so the magnitude of evaluation is sub-second; when the trust is renewed, the real-time trust only needs to be calculated one by one for the number of network agents $n_{N - agent}$ at the expiration of each round of trust credentials, while the number of network agents satisfies the formula (43), and the delay is less than the delay of trust evaluation, and the magnitude is sub-second. When trust is inherited, it only needs to match the users and devices of the network agent, so the delay is also less than 1 ms.

42 $$ \max (n,m) < < k $$

43 $$ n_{N - agent} < n \times m $$

#### Credential verification overhead

Refers to the cost of SDP AH's verification of access credentials provided by SDP IH, that is, using the public key to verify some 16-byte certificates, which takes far less than 1 ms and can be ignored.

#### Component synchronization overhead

Refers to the synchronization delay overhead between the control plane SR PCE/SDN controller and SDP controller, and the synchronization overhead between the two levels of SDP AH in the data plane. Because only the state data needs to be transmitted, this part of the delay is less than the access control delay.

**Figure 8:**
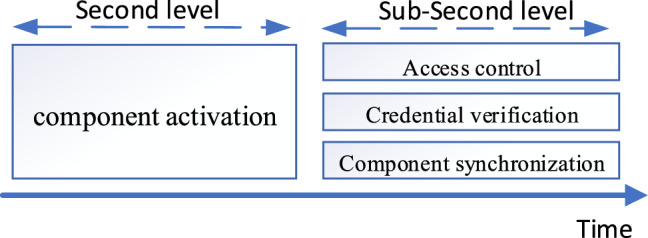
Schematic diagram of the various overhead of SbSR.

As shown in Fig. 8, due to the access control, credential verification, and component synchronization are parallel, the impact of four types of incremental delays in SbSR model on the network depends on the sum of the component startup delay and the maximum delay of the last 3 items in a certain system cycle. According to the above analysis, it is known that the delay is in the order of seconds. In addition, an SDP IH can obtain access credentials of multiple SDP AHs by requesting authorization from the SDP controller once, and one SDP AH can be used to protect multiple service resources, that is, after SDP IH obtains the access rights of an SDP AH, it can use the same access ticket to access multiple resources, which avoids more overhead, so the security overhead brought by SbSR is acceptable.

## Simulation test and analysis

### Simulation environment

**Figure 9:**
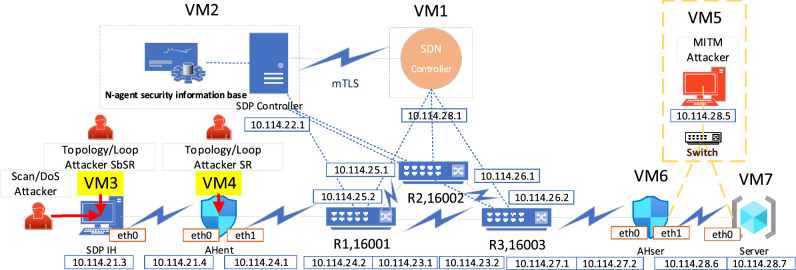
Experimental topology.

EVE-NG (version 3.0.1-16-Pro) is adopted to build the simulation environment. In the control plane, OpenDaylight open-source SDN controller (version Phosphorus 15.0.0) and open-source SDP controller provided by Waverley Labs are respectively deployed in virtual machines VM1 and VM2, in addition, MySQL 5.7.35 and OpenSSL 1.1 software matching SDP controller are installed in VM2. In the data plane, the fwknop module (used to implement SPA authorization protocol) is set in virtual machine VM3 as SDP IH, fwknop module and iptables 1.8 firewall are set in virtual machines VM4 and VM6 as SDP AH_ent_ and SDP AH_ser_ respectively, virtual machine VM5 is set as MITM attacker, and virtual machine VM7 with Web service is set as a service resource. The switching network is composed of SR routers R1, R2, and R3, which are directly interconnected with other virtual machines in the EVE-NG simulation topology, their SIDs are configured as 16,001, 16,002, and 16,003. The trust management mechanism is implemented based on the SDP controller and other open-source components. The trust threshold is set as 0.5 for SDP AH_ent_, and the trust threshold is set as 0.8 for SDP AH_ser_. The Iperf3.1 tool is used to inject network background traffic slowly at an average rate of 1 Mbps. The experimental environment configuration is shown in Table 4, and the topology is shown in Fig. 9, in which the IP of R1, R2 and R3 interfaces with the control plane network element can be known by referring to the IP of the control plane network element, which has been omitted appropriately, and the VM5, Switch and links marked by the yellow dotted line are used for testing, which are not enabled by default.

**Table 4:** Configuration of the experimental environment.

Terminal	OS	CPU	Internal storage	Network adapter/interface	Function
The host machine	Ubuntu 18.04	Intel Core™ i7-7700	32 GB DDR4	Intel(R) Ethernet Connection (7) 1219-V	Host the EVE-NG simulation environment
VM1		–	1 GB RAM	4 NICs	SDN Controller
VM2			1 GB RAM	4 NICs	SDP Controller
VM3			1 GB RAM	2 NICs	SDP IH
VM4			1 GB RAM	2 NICs	SDP AH_ent_
VM5			1 GB RAM	2 NICs	MITM Attacker
VM6			1 GB RAM	2 NICs	SDP AH_ser_
VM7			1 GB RAM	2 NICs	Web Server

### Safety function and performance overhead test

In terms of security function tests, the SDP components in the experimental topology are disabled and enabled respectively to compare the SbSR scheme with the baseline SR scheme to test the security functions of the proposed architecture. The first is port scanning test, namely scanning the service port of VM7 based on VM3 by the hping3 3.0.0 tool.

Second, traffic monitoring test, cut off the direct link of VM6 and VM7, and enable VM5, Switch and communication links marked by the yellow dotted line, then launch a man-in-the-middle attack based on VM5, which is in the same subnet as VM6 and VM7, namely tampering with the traffic path through ARP spoofing to monitor the communication traffic between the two. Third, DoS attack test. Set the bandwidth upper limit to 10Mbps for the virtual interface eth0 of the service resource VM7 and set the bandwidth upper limit of eth1 of VM6 to the same to monitor abnormal traffic rate. According to formula (16), if the attack traffic is greater than 18Mbps, it will constitute a DoS attack. To compare the defense effects of SbSR architecture against DoS attackers of different credit types, VM3 is granted initial access and regarded as attacker A., then cancel its initial access permission and regard it as attacker B; in addition, to demonstrate the defense function of VM6 as SDP AH, it is assumed that VM4 has been compromised and becomes a common host. Then, based on VM3 as attacker A and attacker B, the hping3 3.0.0 tool is used to attack VM7 with 10-s SYN Flood attacks respectively. The two options of "− d 200" and "– Flood" are used in the attack command to send packets with the size of 200 bytes as soon as possible. The OFTest tool is used to test the flow rate based on VM6 and VM7 respectively. Fourth, topology detection test. Using the XCAP tool, by constructing a probe packet with MPLS header label starting from 17 and increasing by 1, TTL = 1 and ICMP header as “Echo Request”, the label and topology of network devices directly connected (the first hop) in the domain are exhaustively detected, and more label and topology probes are carried out in order of hop count and TTL increasing. In the baseline SR scene, the topology in the SR domain is detected based on VM4 which does not enable SDP AH service (VM4 is only regarded as the host at this time); in the SbSR scenario, the attacker sends probe messages based on the authorized SDP IH (VM3). Fifth, routing loop attack topology test. Based on router labels obtained from topology detection, the XCAP tool is used to construct loop attack packets with label stacks {16,002, 16,003, 16,001, 16,002, 16,003, 16,001}, and loop attacks based on directional labels are carried out in the network. In the baseline SR scenario, the attack is launched based on VM4 without the SDP AH function; in the SbSR scenario, the attacker sends attack packets based on the authorized SDP IH (VM3). Due to routing loop attacks need to be detected based on changes in available bandwidth, the Iperf tool is used to detect changes in available bandwidth and Wireshark is used to verify whether there are loop packets in the network or not.

In terms of security overhead test, according to Fig. 8, the security overhead of the SbSR architecture mainly brings more startup delay. Therefore, the network based on the SbSR architecture is compared with the baseline SR network for 10 times of delay tests. Calculate the delay overhead from network startup to the normal operation of all network components, that is, the startup and verification time required by SDP IH to access service resources.

### Safety function test results and analysis

#### Port scanning

**Figure 10:**
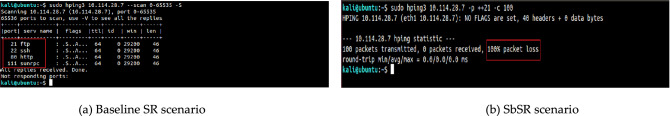
Port scan test (**a**) Baseline SR scenario (**b**) SbSR scenario

As shown in Fig. 10a, in the baseline SR scenario, the scan finds that VM7 opened ports 21, 22, 80, and 111, demonstrating that there is no protection against port scanning at this time, which enabled VM3 to successfully scan ports on VM7. In the SbSR scenario, the result is shown in Fig. 10b, and there is no response. This is because VM3 polls and scans each port frequently in the scanning process. According to the trust penalty function specified in formula (32), trust of VM3 drops below the SDP AH trust threshold, and SDP AH will discard all scan packets received by default. Therefore, VM3 cannot scan for network resource port information of VM7.

#### Traffic monitoring based on a man-in-the-middle attack

**Figure 11:**
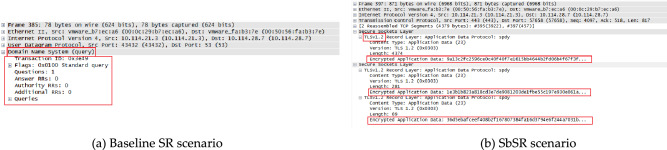
Traffic monitoring test.

By using Wireshark to capture packets, in the baseline SR scenario, the result is shown in Fig. 11a, it is found that the DNS messages between VM6 and VM7 were in clear text. At this time, VM5 could directly obtain the effective communication content between them without decoding, because no encrypted connection is realized between VM6 and VM7. In the SbSR scenario, as shown in Fig. 11b, the communication content between VM6 and VM7 is carried by TCP packets and has been encrypted. It is difficult for VM5 to eavesdrop on VM6 and VM7 based on MITM attack. This is because the mTLS encrypted connection has been established between SDP AH and VM7. Middleman VM5 is unable to decipher the encrypted messages.

#### DoS attack

**Figure 12:**
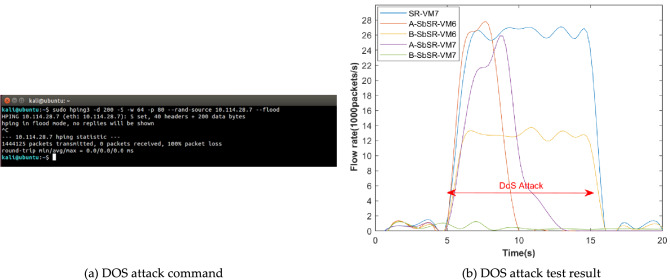
DoS attack test.

The attack command is shown in Fig. 12a. It can be seen that the average packet sending rate of the DoS attacker is about 14412packets / s, that is, the bandwidth occupied is 22Mbps, which meets the DoS attack conditions according to formula (16). The test results based on the baseline SR and SbSR scenarios are shown in Fig. 12b. The blue line indicates that in the baseline SR scheme, the traffic rate based on VM7 increases rapidly due to attacks, and the number of received packets is twice as many as the number of sent packets. This is because the captured streams include SYN attack packets and ACK response packets replied by VM7. The red line indicates that in SbSR scheme, for attacker A, the rate of traffic captured based on VM6(SDP AH) increases rapidly, and the captured traffic also includes SYN attack packets and ACK response packets. However, after the SbSR model detects the DoS attack, VM3 quickly lost its trust and cannot continue to transmit attack traffic. The purple line is the traffic rate captured by the eth1 interface based on VM7, which is basically the same as the red line but slightly delayed. This is because the packet type captured on VM7 is basically the same as that on VM6, but slightly delayed for the start and end of attacks on VM3 due to the ethernet interface buffer size. The yellow line indicates that in SbSR scheme, for attacker B, because it does not obtain access permission, VM6 adopts the "drop-all" policy by default and discards all SYN attack packets sent by it. VM7 does not generate ACK packets for attack packets, and only the SYN packets sent by the attacker are captured based on VM6 interfaces. At this time, ACK packets account for about half of the total traffic, so the flow rate measured based on VM6 is halved. The green line represents the traffic captured by VM7, due to VM6 discards all attack traffic by default, leaving only a small amount of network background traffic with a traffic rate of basically 0 in VM7.

#### Topology detection based on label detection

**Figure 13:**
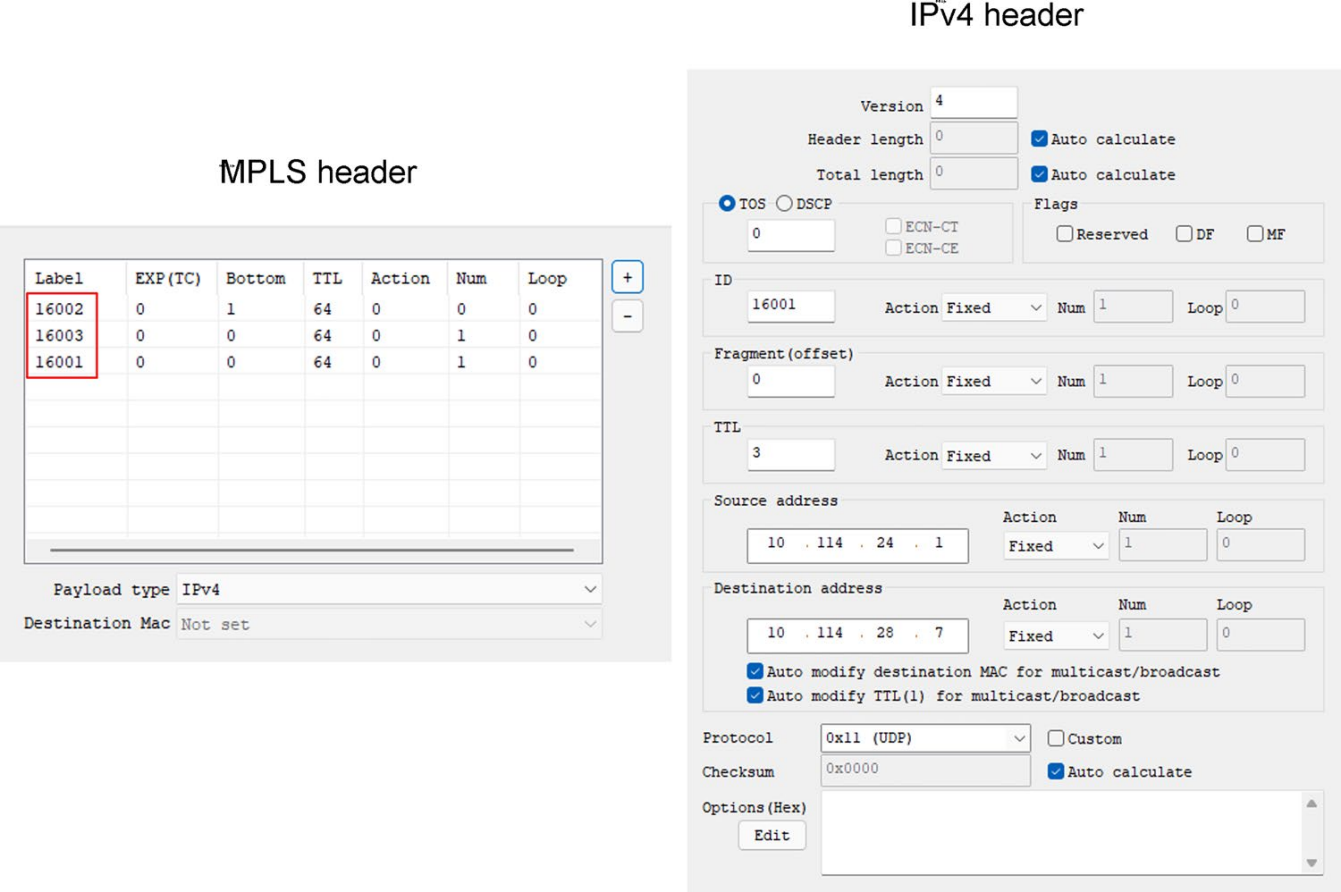
Topology probe attack packet.

The attack packet settings are as shown in Fig. 13. In the baseline SR scenario, the ICMP timeout message can be found through packet grabbing. Because the ID field of the detection message is synchronously traversed with the SR label value, the label value of the next-hop can be judged according to the IPv4 header ID field, and according to this, it is successfully detected that the labels of the two nodes directly connected to the domain entry node R1 are 16,002 and 16,003 respectively, and then the label of R1 itself is detected as 16,001. This is because no real-time trust control mechanism has been established for access devices, resulting in the hop-by-hop traversal detection of the intra-domain topology by the host VM4 can be successfully realized. In the SbSR scenario, ICMP messages fail to be captured. The reason is that the labels of packets sent by the SDP AH are increasing, and the trust value of the packets decreases rapidly. As a result, access of VM4 is denied and detection attacks are blocked.

#### Routing loop attack based on directional label

**Figure 14:**
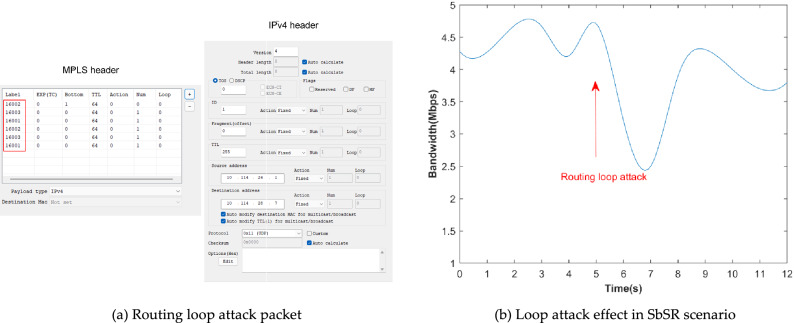
Routing loop attack test.

Attack packet Settings are shown in Fig. 14a. In the baseline SR scenario, the Iperf tool is used to measure the available bandwidth. The available bandwidth decreases after the attack starts. The Wireshark tool is used to capture packets and the result show that the packets are running continuously in the network and occupy certain bandwidth due to loops. In the SbSR scenario, the available network bandwidth measured by Iperf tool is shown in Fig. 14(b). It can be seen that the average available network bandwidth decreases by about 47.6% after the loop attack starts at the 5th second, until it recovers at about 8.4 s. This is because SDP AH enables the trust penalty mechanism for SDP IH according to formula (32) after detecting the routing loop, resulting in the invalid access credentials of VM3 and the deletion of the imported routing loop packet.

### Performance overhead test results and analysis

**Figure 15:**
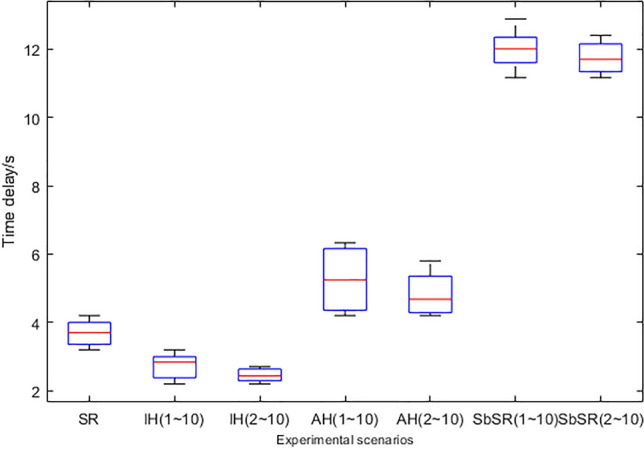
Comparison of time delay in different scenarios.

The delay numerical distribution box figure is shown in Fig. 15. Based on the data distribution of 1–10 experiments, it can be seen that the average startup delay increment introduced by the SDP component in SR network is 8.31 s, which is a one-time delay generated during startup. And the introduction of new SDP IH and SDP AH will introduce 2.84 s and 5.25 s of average startup verification delay respectively. Based on the data distribution of 2–10 experiments, it can be seen that for SDP IH and SDP AH with network records, the average delay decreases to 2.44 s and 4.68 s respectively, and the average increment of total delay decreases to 8 s. This is because SDP IH and SDP AH rapidly improve their trust value according to the trust inheritance mechanism specified in formula (35) and (39). In fact, because the deployment of SDP AH is relatively fixed, the main impact of the introduction of the SbSR architecture on network service performance is the introduction of an average startup and validation delay of about 2.84 s each time a new network agent is connected, which is acceptable compared to the average failure time of the network caused by attacks. At the same time, this delay is directly related to the trust management mechanism and can be further reduced by optimization of related algorithms.

From the perspective of algorithm complexity, based on the analysis of the mechanism overhead composition and magnitude in “SbSR overhead analysis” section, assuming that the number of network agents in the network is $n_{N - agent}$, the network agent enable speed is a fixed value $v_{e}$, and the calculation amount of each trust evaluation and trust renewal of a network agent is a fixed value $k$, the evaluation interval is $t$, and the network agents are enabled one by one (not simultaneously). At this time, the main performance overhead of SbSR is concentrated in the component enablement overhead (second level), followed by the trust evaluation and trust renewal cost in the access control overhead (sub-second level). The component enablement overhead has nothing to do with the algorithm of the SbSR mechanism itself, and it will introduce a time cost with a time complexity of *O*($\frac{{n_{N - agent} }}{{v_{e} }}$), resulting in a significant increase in network time delay, therefore, the 2.84 s average startup verification delay introduced by new network agent is mainly composed of the component enablement delay; for the latter overhead, since the trust calculation time is extremely short and frequent, the calculation overhead with time complexity of *O*($\frac{{kn_{N - agent} }}{t}$) will be mainly introduced, and has a lower latency impact on the network. Therefore, the time overhead of the SbSR model is related to the number of network agents enabled one by one, and the computational cost is related to the trust evaluation interval, the calculation amount of proposed algorithm, and the number of network agents.

## Conclusion and future work

Segment Routing technology has been proved by practice to be a better implementation form of SDN, but its data plane is faced with many security problems such as data tampering, malicious access, denial of service attack, and so on. For the SR data plane, this paper proposes a security model based on SDP trust enhancement architecture. By setting mechanisms such as initial trust grant, evaluation, renewal, and inheritance for SR data plane access devices, the proposed model can evaluate and control their trust value in a process. After 6 kinds of security functions and cost tests, it is proved that the proposed model can improve the security performance of the SR network data plane to a certain extent with affordable delay cost, indicating that the introduction of the SDP architecture may provide better security performance for the existing network, and this security solution is worth being applied to other scenarios. At the same time, by reviewing the model design and test results, it can be seen that the method and indicators used by the model proposed in this paper to grant initial trust values ​​and evaluate real-time trust may not be perfect enough to deal with other new threats, and cannot detect malicious routing devices in the network domain. Next, we will focus on the initial trust grant mechanism and real-time trust evaluation mechanism, relevant verification, and evaluation indexes and algorithms will be improved, and the trust management mechanism for routing devices will be supplemented and improved.
